# On a Simple Mode of Reducing a Dislocated Elbow

**Published:** 1857-05

**Authors:** 


### *On a Simple Mode of Reducing a Dislocated Elbow.* By M. Bidard, of Arras.

In a recent communication to the Société de Chirurgie de Paris, M. Bidard relates a case in which a dislocated elbow was reduced in a very simple manner, after the ordinary means had failed. A child, æt. 13, had dislocated his elbow, and the dislocation had been reduced in the ordinary way. A month later the elbow was again dislocated. On this occasion the child said nothing about the accident, and five weeks passed before the mischief was discovered, and the attempts at reduction repeated. These attempts failed. It then occurred to M. Bidard to persuade the child to swing himself by both his hands from a cross beam of wood, and to allow his hands to be held in this position by another person when he became tired. These swingings were continued for fifteen or twenty minutes at a time, and repeated every morning and evening; and the result was, that the displacement had entirely disappeared on the seventh day. It appears from the account, that the displacement diminished progressing between the first and ninth suspension; and that the rest of the deformity disappeared suddenly during the fourteenth suspension.

This method, as M. Larrey observed afterwards, possesses some analogy to that of the *door*, as formerly practiced by some surgeons, but with this difference, that the reduction is effected gradually in this and suddenly in that,

As to the rest, we are disposed to think there need have been no difficulty if chloroform had been employed; for, unquestionably, dislocations of much older standing are easily reducible with the help of this agent.—*Gaz. Hebdom. de Méd. et Chir.*

